# Intrarenal Adrenocortical Adenoma Treated by Robotic Partial Nephrectomy with Adrenalectomy

**DOI:** 10.1089/cren.2016.0017

**Published:** 2016-02-01

**Authors:** Samay Sappal, Jay Sulek, Steven C. Smith, Lance J. Hampton

**Affiliations:** ^1^ School of Medicine, Virginia Commonwealth University, Richmond, Virginia.; ^2^ Division of Urology, Virginia Commonwealth University, Richmond, Virginia.; ^3^ Department of Pathology, Virginia Commonwealth University, Richmond, Virginia.

## Abstract

***Background:*** We present an intrarenal adrenocortical adenoma discovered incidentally after robot-assisted partial nephrectomy and total adrenalectomy for a suspicious renal mass. Current literature describes the rare occurrence of an adrenocortical adenoma arising from a renal–adrenal fusion. This case represents an uncommon, benign pathology that should be considered in the differential diagnosis of an enhancing renal mass.

***Case Presentation:*** The patient is a 62-year-old female found to have an enhancing mass at the anterolateral aspect of the upper pole of the right kidney concerning for renal-cell carcinoma. CT imaging was performed to work up a cause for hyperparathyroidism. During robot-assisted partial nephrectomy, the lesion was found to be partially adherent to the lateral limb of the right adrenal gland. Microscopic evaluation with Melan-A staining showed the mass to be of adrenal origin with benign features and lack of capsulation, indicating an adrenal adenoma arising from intrarenal ectopic adrenal rests.

***Conclusion:*** An intrarenal adrenal adenoma arising from ectopic adrenal tissue is a unique pathology that represents a benign differential diagnosis in the evaluation of an enhancing renal mass. However, it cannot be differentiated from renal-cell carcinoma based on cross-sectional imaging alone and requires postoperative pathologic assessment to confirm the diagnosis.

## Introduction and Background

The occurrence of intrarenal adrenocortical adenoma is exceptionally rare. One study conducted for more than 13 years at Johns Hopkins University found only one case of a cortical adenoma arising within the renal parenchyma.^1^ On CT, an intrarenal adenoma may appear as a solid enhancing renal mass and may lead to the presumptive diagnosis of renal-cell carcinoma.^2^ In general, such a case will not be discovered to be benign until pathologic analysis of the surgical specimen is conducted after partial or radical nephrectomy. Moreover, biopsy of such a lesion poses important diagnostic challenges given both the possibility of an adrenocortical neoplasm to simulate clear cell renal-cell carcinoma^1^ and the potential to interpret a sample of unexpected adrenocortical tissue as a nondiagnostic sample of tissue adjacent to a lesion.

Here we present the case of a 2.7-cm right upper pole renal mass that was incidentally discovered after CT was performed to work up a cause for hyperparathyroidism. Robotic partial nephrectomy and adrenalectomy were performed for a suspected renal-cell carcinoma with pathologic analysis revealing a benign intrarenal adrenocortical adenoma arising from rests of ectopic adrenal tissue in the superior pole of the kidney.

## Presentation of Case

**FIG. 1.**
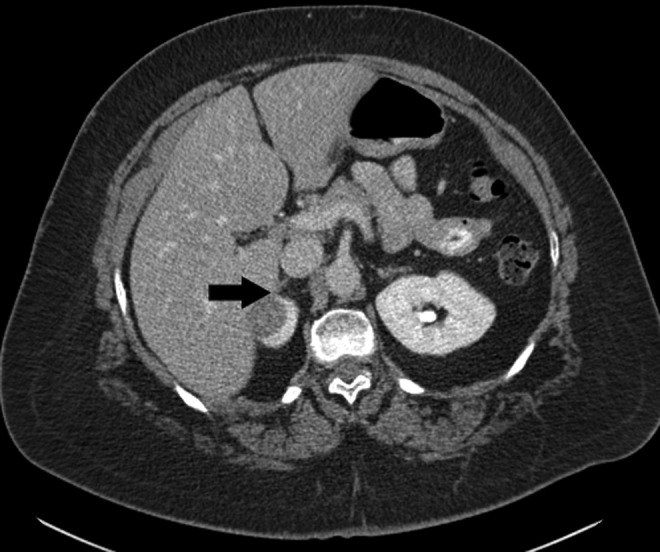
CT image. Transverse cut in cortical phase. An enhancing renal mass (*black arrow*) is seen at the upper pole of the right kidney and is abutting the lateral limb of the right adrenal gland.

A 62-year-old female presented after the incidental finding of a renal mass. The patient had a history of multinodular goiter with hypercalcemia, vitamin D deficiency, and osteoporosis for which she had received a hemithyroidectomy and levothyroxine replacement therapy. The patient denied flank pain or hematuria. There were no pertinent findings on physical examination. A basic metabolic panel, hepatic panel, and calcium level were all unremarkable, and hemoglobin was within normal limits. Parathyroid hormone was elevated at 125.5 pg/mL. We obtained a CT renal mass protocol, which revealed a largely exophytic 2.7 cm enhancing mass at the anterolateral aspect of the upper pole of the right kidney (Fig. 1). Of note, the lesion appeared to be partially inseparable from the right adrenal gland and directly abutting the liver. The patient elected to undergo right robot-assisted partial nephrectomy with possible adrenalectomy.

Intraoperatively, the lesion was found to be adherent to the lateral limb of the right adrenal gland. The lesion appeared to bulge down onto the renal parenchyma and extend into the adjacent adrenal. Consequently, an adrenalectomy was performed and the lesion was sent for pathologic evaluation. The procedure was otherwise uncomplicated with a warm ischemia time of 14 minutes and a total operation time of 2 hours and 10 minutes. The patient was discharged home on postoperative day 1.

**FIG. 2.**
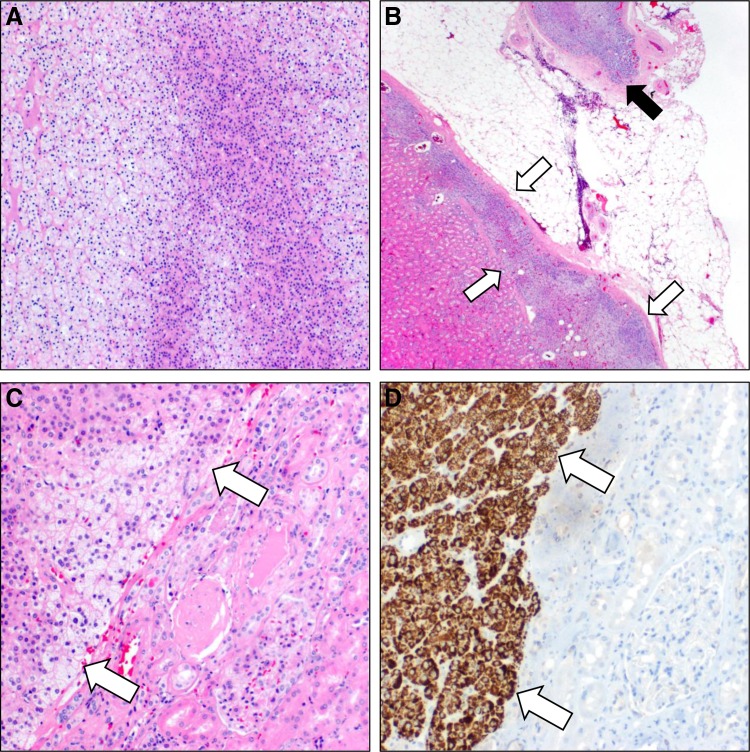
Pathologic findings establishing diagnosis. **(A)** The tumors were composed of solid nests of plump cells with variable clear and eosinophilic cytoplasm without atypia, necrosis, or mitosis. **(B)** Adjacent to the tumor, a plaque-like area of intrarenal adrenal rests is seen (*white arrows*) unassociated with the adjacent adrenal gland (*black arrow*). **(C)** No capsule is seen between the adrenocortical adenoma (*white arrows*) and adjacent kidney. **(D)** Confirmatory of adrenal origin, Melan-A is diffusely positive in the adenoma tissue (*white arrows*) but not in the adjacent normal renal tubules.

The final pathology report grossly described a partial nephrectomy specimen, overall 4.5 cm, with 3.1 cm of attached adrenal gland in perinephric fat. Sections showed a 2.7 cm mottled brown-yellow nodule in the kidney, protruding up to 0.7 cm outward from the surface, closely approaching the attached adrenal gland, but it was unclear grossly whether it was involved. On histologic sections, the solid nodule consisted of variably clear and eosinophilic cells with nested growth and without atypia, necrosis, or invasion, supportive of classification as an adenoma (Fig. 2A). In the renal parenchyma adjacent to the lesion were aberrant intrarenal adrenal rests, visibly separated from the adjacent, otherwise normal adrenal gland (Fig. 2B). Supportive of the adenoma arising from the intrarenal adrenal rests, there was a lack of encapsulation at the interface with the normal kidney (Fig. 2C). Confirmatory of adrenal origin and excluding a primary renal neoplasm, the tumor was diffusely positive by immunohistochemistry for Melan-A, an adrenal marker (Fig. 2D), and negative for PAX8, a sensitive marker of renal origin.

## Discussion and Literature Review

Intrarenal ectopic adrenal tissue is the presence of adrenal heterotopia within the renal parenchyma. Microscopically, it appears as a mixture of renal tubular and cortical clear cells with a lack of capsulation. Rokitansky first described the phenomenon of renal–adrenal fusion, in which adrenal tissue arises within the renal parenchyma developmentally or secondary to postinflammatory fibrosis of perirenal fat.^1^ Although the developmental pathogenesis of renal–adrenal fusion has not been clarified, it is proposed that it is due to the failure of capsule formation from the retroperitoneal mesenchyme during organogenesis.^3^ Considered distinctly different is the observation of ectopic adrenal tissue within the renal parenchyma, which is seen with some frequency in newborns, involuting in infancy, and apparent in ∼1% of adults.^4^ Adrenal ectopy is thought to occur when a portion of the primordial adrenal gland becomes detached during development and becomes lodged in other developing viscera, including the kidney. Depending on when this occurs, it may consist of either only cortical tissue or cortical and medullary tissue, as reviewed recently.^1^ Such rests generally do not come to light, unless, rarely, a neoplasm arises from them, as observed here.

The literature reveals few occurrences of intrarenal adrenal heterotopia.^1^ Thus, even more infrequent is the development of a benign adrenal adenoma from the ectopic tissue. We note that one case was reported to have arisen from a renal–adrenal fusion.^2^ Whether the scenario involves renal–adrenal fusion or a neoplasm arising in ectopic adrenal rests as seen here, such a case requires close communication between surgery, radiology, and surgical pathology. Although contemporary stains can easily distinguish adrenal from renal origin, the propensity for adrenocortical adenoma to show clear cell morphology could result in incorrect diagnosis if suspicion is not conveyed. Given the rarity of this presentation, the excellent visualization afforded by robot-assisted partial nephrectomy in this case was important in establishing the close relationship with the adrenal gland.

## Conclusion

We report a case of a right-sided enhancing upper pole renal mass found incidentally on CT for work up of a patient with hyperparathyroidism that was suspected to be renal-cell carcinoma. During robot-assisted partial nephrectomy, the lesion was found to be inseparable from the lateral limb of the right adrenal gland and pathologic evaluation confirmed the rare occurrence of an adrenal cortical adenoma arising from aberrant intrarenal adrenal rests. The use of the robotic platform allowed this rare mass to be removed in a time-efficient and nephron-sparing approach with minimal warm ischemia time. Although current cross-sectional imaging modalities are unable to differentiate this type of lesion from renal-cell carcinoma and only postoperative assessment of the excised mass can distinguish it as being benign, it is important to consider this rare pathology in the differential diagnosis of an enhancing renal mass.

## Abbreviation Used

CT: computed tomography

## References

[1] Ye H, Yoon GS, Epstein JI Intrarenal ectopic adrenal tissue and renal-adrenal fusion: A report of nine cases. Mod Pathol 2009;22:175–181 18820668 10.1038/modpathol.2008.162

[2] Mahadevia S, Rozenblit A, Milikow D, et al. Renal-adrenal fusion: Instance of an adrenal adenoma mimicking a solid renal mass at CT—Case report. Radiology 2009;251:808–812 19261923 10.1148/radiol.2511081151

[3] Honore LH, O'Hara KE Combined adrenorenal fusion and adrenohepatic adhesion: A case report with review of the literature and discussion of pathogenesis. J Urol 1976;115:323–325 1255896 10.1016/s0022-5347(17)59188-0

[4] Souverijns G, Peene P, Keuleers H, et al. Ectopic localisation of adrenal cortex. Eur Radiol 2000;10:1165–1168 11003415 10.1007/s003309900263

